# Stress Induced Mitochondrial Hyperfusion (SIMH): A Concise Review on SIMH in Neurodegenerative Disorders and Other Diseases

**DOI:** 10.61747/0ifp.202503002

**Published:** 2025-06-05

**Authors:** Jeffrey D. Brown, Mei-zhen Cui, Xuemin Xu

**Affiliations:** ‡ Department of Biology, College of Art and Science, The University of Texas Permian Basin, Odessa Texas 79762

**Keywords:** SIMH, Mitochondrial dynamics, Neurodegenerative disorders, ER Stress, MAM, UPR, Mfn, OPA1, Drp1

## Abstract

Mitochondria are critical for cell health, and damaged or dysfunctional mitochondria have been strongly linked to various human diseases, particularly neurodegenerative disorders. Mitochondrial function is regulated by several mechanisms, including the regulation of mitochondrial shape, size, number, and morphology. Mitochondria constantly fuse together and separate; this fusion and fission process is known as mitochondrial dynamics. These mitochondrial dynamics are modulated in response to various stimuli, and recent reports have demonstrated that mitochondria undergo hyperfusion under mild to moderate stress conditions, resulting in the formation of elongated filaments. This phenomenon is referred to as stress induced mitochondrial hyperfusion (SIMH). SIMH is associated with enhanced protection of mitochondria and improved cell viability under stress conditions. The induction of hyperfusion can be triggered by diverse stressors, each with distinct and unique mechanisms. This mini review will focus on these stressors and their corresponding mechanisms, as well as the subsequent effects on mitochondrial and cell health. Additionally, disease models which demonstrate a correlation between specific disease-related stress conditions and mitochondrial hyperfusion are discussed.

## INTRODUCTION & BACKGROUND: MITOCHONDRIAL DYNAMICS

**Figure 1.**
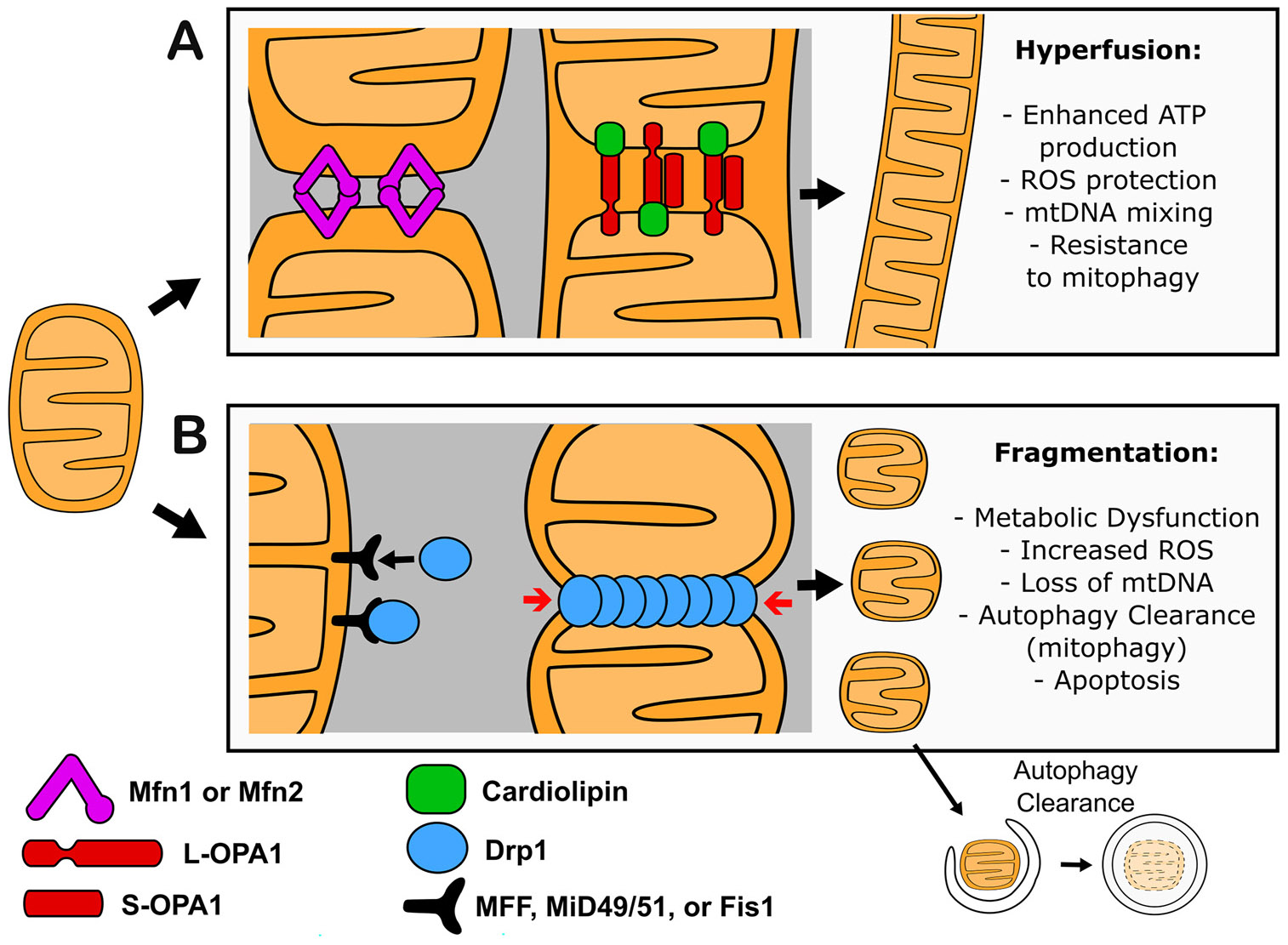
Mechanisms of mitochondria fusion and fission Mitochondrial fusion and fission work together to maintain mitochondrial shape and size in a process known as mitochondrial dynamics. [1]. Mitochondrial dynamics are highly regulated and vital for maintaining mitochondrial homeostasis and regulating mitochondrial function. Imbalances between fusion and fission result in either hyperfused or fragmented mitochondria, each of which can have drastic effects on various mitochondrial functions such as metabolism, mitochondrial quality control, ROS production, and cell health (right). **(A)** Mitochondrial fusion takes place in two steps, starting with the outer mitochondrial membrane (OMM) fusion, followed by fusion of the inner mitochondrial membrane (IMM). OMM fusion is mediated by the proteins Mfn1 and Mfn2 (purple), while IMM fusion is mediated by isoforms of the protein OPA1 (red) and the lipid Cardiolipin (green). **(B)** Mitochondrial fission is mediated by Drp1 (blue) and the OMM Drp1receptor proteins MFF, MiD49, MiD51, and/or Fis1 (black). Following recruitment onto the OMM, Drp1 forms a ring-like structure which constricts the mitochondria. The functions of Mfn1, Mfn2, OPA1, and Drp1 all depend on their GTP hydrolysis activity as they are all GTPases.

Mitochondria maintain their shape, size, and function by constantly merging together, a process known as fusion, and separating a process known as fission. These processes are commonly referred to as mitochondrial dynamics [1], which are highly regulated and crucial for maintaining mitochondrial homeostasis and facilitating energy adaptation (Figure 1). Mitochondrial dynamics are also important for various regulatory and physiological functions, including metabolism, endoplasmic reticulum (ER) signaling, calcium homeostasis, proteostasis, autophagy-mediated turnover of mitochondria, and apoptosis (reviewed by [2–5]). The regulation of mitochondrial dynamics are currently known to be governed by a subset of GTPases within the dynamin family of proteins; however, this is an intricate regulatory mechanism which is not yet fully elucidated (for review see [1]). Briefly, in mammalian cells, three GTPase proteins are reportedly involved in mitochondrial fusion: mitofusin 1 and 2 (Mfn1 and Mfn2) and optic atrophy 1 (OPA1). In contrast, only one GTPase protein is believed to mediate mitochondrial fission: dynamin related/-like protein 1 (Drp1) (Figure 1).

Mfn1 and Mfn2 are localized to the outer mitochondrial membrane (OMM), where they facilitate fusion of the outer membrane of adjacent mitochondria by forming homo- or hetero-dimers with Mfns on neighboring mitochondria [6] (Figure 1). Both Mfn1 and Mfn2 have been reported to play a pivotal role in tethering and fusing of OMMs. However, it has been observed that Mfn1 exhibits superior GTPase and tethering activities compared to Mfn2. Consequently, Mfn1 is regarded as the primary GTP-dependent membrane tethering protein for facilitating mitochondrial fusion [7]. This notion is further supported by the fact that Mfn2, in addition to its location on the mitochondria, is also found in the endoplasmic reticulum (ER). On the ER, Mfn2 interacts with either Mfn1 or Mfn2 on the mitochondrial surface to establish tethering between these two organelles, leading to the formation of specialized cellular compartments known as mitochondria associated membranes (MAMs) [8].

The protein OPA1 is involved in the process of inner mitochondrial membrane (IMM) fusion and maintenance of cristae structure. The human OPA1 is characterized by eight isoforms, resulting from alternative splicing, and processing at two proteolytic cleavage sites, termed S1 and S2, resulting in at least 5 bands detectable by western blot. The two heaviest bands (termed long OPA1 or L-OPA1) anchor on the IMM, while processing at the S1 site by the metalloprotease OMA1 and/or at the S2 site by the metalloprotease YME1L lead to the formation of distinct, unanchored forms of short OPA1 (S-OPA1), depending on the isoforms [9]. L-OPA1 alone is sufficient to drive IMM fusion. Further, OPA1 is only required on one of the two fusing IMMs, as heterotypic interaction between OPA1 and the lipid cardiolipin on opposing membranes is sufficient to drive fusion (Figure 1). Though cleavage at the S1 and S2 sites has been shown to effect both IMM-specific fusion and mitochondrial fusion as a whole, how the balance between LOPA1 and S-OPA1 contributes to mitochondrial dynamics is not well understood, as many previous studies have been inconsistent [1].

For both the IMM and the OMM, mitochondrial fission is driven by Drp1. Drp1 is a cytosolic protein which lacks a mitochondrial membrane binding domain, therefore fission activity is dependent on receptor-mediated Drp1 oligomerization on the OMM (Figure 1). The OMM receptor proteins involved in mitochondrial fission includes mitochondrial fission factor (MFF), mitochondrial dynamics proteins 49 & 51 (MiD49 & MiD51), and mitochondrial fission protein 1 (Fis1)- though more recent studies suggest that Fis1 plays only a minor role [1]. Drp1 is regulated by a variety of post-translational modifications, with most studies focusing on either fission-inhibiting phosphorylation at Ser637 or pro-fission phosphorylation at Ser616 [1]. While it has been assumed that Drp1-induced constricting forces are solely responsible for final membrane scission events, this notion has been challenged by a recent study, which highlights the essential role of Dnm2, another member of the conventional dynamin family, in these processes [10].

As mitochondrial dynamics play a crucial role in maintaining mitochondrial function, the dysregulation of mitochondrial dynamics has been strongly implicated in various neurodegenerative diseases (NDs) (reviewed by [5]). Both excessive fission and fusion are associated with distinct aspects of cell health status. In general, in addition to mtDNA replication [11] and mitochondrial redistribution during cell division [12], as a result of fission, fragmented mitochondria are associated with metabolic dysfunction, selective sequestering and recycling of dysfunctional mitochondria (mitophagy), increased production of reactive oxygen species (ROS), loss of mitochondrial DNA (mtDNA), and apoptosis [2–5,13]. On the other hand, the process of mitochondrial fusion dilutes the existing damage and facilitates the exchange of functional components among mitochondria [14]. Consequently, elongated, tubular mitochondria are commonly associated with enhanced ATP production, protection against ROS damage, preservation of intact mtDNA through mixing mechanisms, and resistance to mitophagy (see Figure 1). [1–5,15,16].

The rates of fission-fusion dynamics are commonly regulated by the metabolic and pathogenic states of organelles and cells, while overall equilibrium is usually maintained. Studies have demonstrated that mitochondria can respond to stress by altering their fission and fusion dynamics [14] and under cellular stress, the balance of mitochondrial dynamics shifts towards fusion as a response to such stress. The excessive fusion leads to the formation of elongated and hyperfused mitochondria through a process known as stress-induced mitochondrial hyperfusion (SIMH) [17]. The induction of SIMH has been demonstrated by various stressors, including starvation, disruption of proteostasis, oxidative stress, and DNA damage. Several of these SIMH-inducing stressors have been shown to strongly associate with neurodegenerative diseases, thereby suggesting a potential suitability of modulating these responses for future therapeutic interventions. These different stressors induce SIMH through distinct signaling pathways, thereby exerting diverse effects on cellular function. The aim of this mini review is to provide a concise and well-organized overview of the different physiological insults that have been demonstrated thus far to induce SIMH, with a particular emphasis on their relevance to ND. Lastly, several other disease models are discussed which have shown a correlation between mitochondrial hyperfusion and phenotypic outcomes.

## ER-MITOCHONDRIA CONTACT SITES (ERMCS) AND MITOCHONDRIAL HYPERFUSION

The endoplasmic reticulum (ER) and mitochondria form physical contact points known as ER-Mitochondria contact sites (ERMCS), or mitochondria-associated endoplasmic reticulum membranes (MAMs), which play an indispensable role in various cellular processes. [18]. To determine the mitochondrial division or fission sites, recent study revealed that dynamin-related proteins involved in mitochondrial division localize to regions of MAM(ERMCS) and that these regions are associated with constricted mitochondria and subsequent division, implying a direct involvement of the ER in the process of mitochondrial division [19]. Specifically, the discovery that ER-mitochondrial contacts independently establish positions of mitochondrial constriction, irrespective of Mff and Drp1 recruitment, suggests that ER-mediated mitochondrial fission occurs through physical wrapping around the mitochondria resulting in pre-constriction followed by DRP1 recruitment and division [19]. Hence compromised interactions between these two organelles may impact hyperfusion by inhibiting mitochondrial fission and altering the balance of mitochondrial fission-fusion towards excessive fusion (Figure 1). The involvement of MAM in mitochondrial fusion is further supported by the fact that Mfn2, in addition to its location on the mitochondria, is also found in the ER. On the ER, Mfn2 interacts with either Mfn1 or Mfn2 on the mitochondrial surface to establish tethering between these two organelles, leading to the formation of specialized cellular compartments MAM [8,20]. Furthermore, mitochondrial fusion and elongation is promoted by knockdown of trichoplein, which binds to ER-anchored MFN2 and disturbs ER-mitochondria interaction [21]. In addition, as discussed below, during ER stress, the MAM-resident PERK activation triggers mitochondrial hyperfusion, thereby preventing premature apoptotic fragmentation of the mitochondria [22].

## PROTEOSTASIS & ER-STRESS-INDUCED SIMH

### Dysregulation of Protein Synthesis

One of the first reports of SIMH was by the name-sake paper [17], where exposure of various cell types to the RNA synthesis inhibitor actinomycin D, the protein synthesis inhibitor cycloheximide (CHX), or UV-irradiation resulted in the formation of a highly interconnected and slender mitochondrial tubular structure. This stress stimuli-induced change in mitochondrial morphology was referred to as “stress-induced mitochondrial hyperfusion (SIMH)” [17], and several research groups have recapitulated this finding since then [23–25]. Compared to fusionincompetent cells treated with CHX, hyperfused cells exhibit enlarged cristae with a more condensed matrix, resulting in increased ATP production and enhanced resistance against CHXinduced apoptosis (although prolonged exposure still leads to apoptosis) [17,24]. This protective effect likely arises from fusion-dependent activation of the transcription factor NF-κB, which significantly contributes to resistance against apoptosis [24]. This SIMH response is dependent on Mfn1 (but not Mfn2) and the long isoform of OPA1 (L-OPA1), as well as the IMM scaffolding protein SLP-2, which was found to be required for maintenance of L-OPA1 during stress [17]. Though the exact mechanism remains unknown, CHX-induced SIMH is thought to be a result of increased mitochondrial fusion.

A subsequent study revealed that SLP-2 forms complexes with the IMM proteases YME1L and OMA1, which are both implicated in the processing of OPA1 [26], wherein SLP-2 acts as an inhibitor of YME1L and OMA1 to protect L-OPA1 from cleavage by OMA1 and YME1L at the S1 and S2 sites, respectively, thereby facilitating stress-induced fusion [27].

The protein synthesis-inhibition SIMH response is significant as it represents one of the initial reports on SIMH, albeit without substantial implications in neurodegenerative diseases (ND). However, further research is required to establish a direct link between inhibition of gene expression (and subsequent hyperfusion responses) and ND, despite the widespread use of cycloheximide as an autophagy inhibitor [28] and the implication of NF-κB signaling regulation in ND [29].

### Unfolded Protein ER Stress

**Figure 2.**
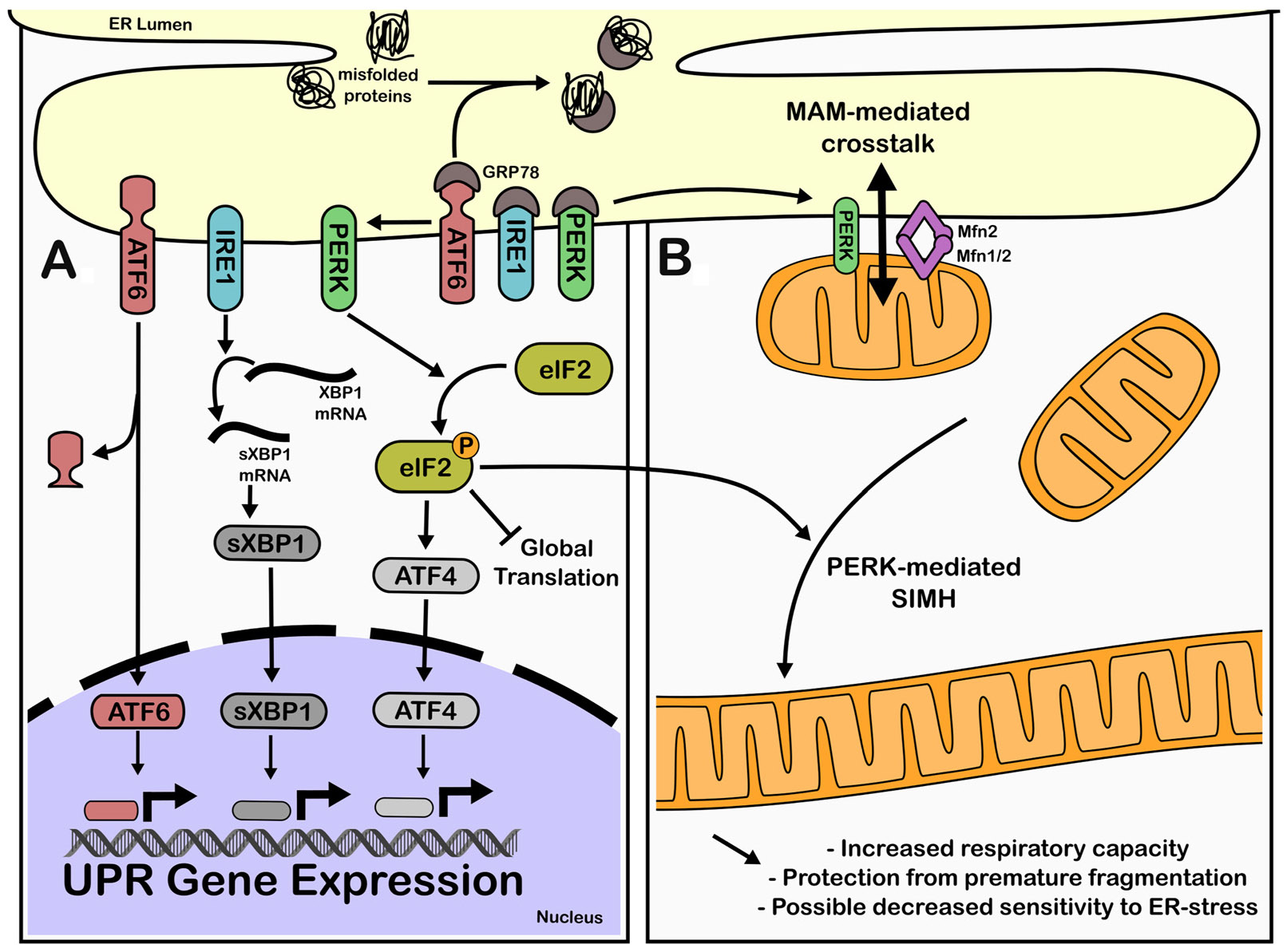
The Unfolded Protein Response (UPR) and stress induced mitochondrial hyperfusion (SIMH) **(A)** Following an ER-stress event, the UPR is initiated through three signaling pathways which work together to reduce protein load and restore proteostasis. These three response pathways are mediated by three ER transmembrane proteins: ATF6 (red), IRE1 (blue), and PERK (green). Under normal physiological conditions these three proteins are kept inactive by the protein GRP78/BiP (grey). However, during ER stress, misfolded proteins accumulate in the ER lumen sequester GRP78, leading to the activation of ATF6, IRE1, and PERK. Upon activation, ATF6 translocates to the Golgi Apparatus (not shown), wherein ATF6 is cleaved. Following cleavage, the resulting cytosolic ATF6 fragment translocates into the nucleus where it functions as a transcription factor of UPR-related genes. IRE1 upon activation cleaves an intron from XBP1-encoding mRNA, which is translated into the frame-shift variant of XBP1 (sXBP1, dark grey), an active transcription factor. PERK, following activation, phosphorylates eIF2 (yellow), which attenuates global translation and subsequently reduces the protein load on the ER. A select group of transcripts are immune to this translation block, permitting their expression following eIF2 phosphorylation. One such transcript encodes the protein ATF4 (light grey), a transcription factor. These three transcription factors (the cytosolic fragment of ATF6, sXBP1, and ATF4) express UPR-related genes which work together to promote protein quality control and restore proteostasis following ER stress [33,34]. **(B)** PERK is unique among these three UPR-mediating proteins in that it also regulates mitochondrial function and works to protect the mitochondria during ER-stress events. PERK is a key structural component of the ER mitochondrial associated membrane (MAM), a tethering site between mitochondria and the ER. Within the MAM, PERK mediates calcium signaling, MAM-mediated apoptotic signaling, mitochondrial sensitivity to ROS-stress, and stronger ER-mitochondrial tethering [36]. Further, PERK mediates ER-stress induced SIMH in a manner dependent on SLP-2 and YME1L (not shown) downstream of eIF2 phosphorylation. This hyperfusion response acts as a protective mechanism which enhances mitochondrial metabolism and inhibits pathological fragmentation following ER-stress [37].

On the other hand, mitochondrial hyperfusion has been demonstrated as a defensive response during endoplasmic reticulum (ER) stress, which is associated with neurodegenerative disease. During this stress, as a protective mechanism, hyperfusion occurs to prevent premature fragmentation of mitochondria and optimize energy production [22]. The ER is a dynamic organelle that plays a pivotal role in various cellular functions including protein synthesis, lipid synthesis, the regulation of intracellular calcium levels, and redox balance. As such, it serves as a crucial organelle involved in maintaining cellular homeostasis [30]. During gene expression, the process of protein folding is vital for the survival of living organisms as it contributes to the structural framework encoded by such genes. Even a slight deviation during folding can result in abnormal conformation, which may have catastrophic consequences. The term “ER stress” refers to physiological or pathological conditions that result in the accumulation of misfolded proteins within the endoplasmic reticulum [31]. ER stress disrupts the normal physiological functions of cells and triggers a cellular response known as the unfolded protein response (UPR), which is an adaptive survival mechanism enabling cells to cope with the stress caused by misfolded proteins [32]. UPR is primarily regulated by three transmembrane proteins located in the ER, namely inositol-requiring enzyme 1 (IRE1), activating transcription factor 6 (ATF6), and protein kinase RNA-like ER kinase (PERK) [33]. As shown in Figure 2, the three ER stress sensors IRE1, ATF6, and PERK are maintained in an inactive state under normal conditions through interactions with the ER chaperone protein GRP78 (BiP). During ER stress, GRP78 is sequestered by accumulated unfolded/misfolded proteins, which leads to the activation of the three stress sensors [34]. Once activated, each of these three sensors triggers distinct signaling pathways that collectively result in a reduction in protein load by inhibiting global protein synthesis and upregulating genes associated with protein folding, quality control, and ER associated degradation. Ultimately, the process restores homeostasis and ER function [33]. (Figure 2).

Interestingly, PERK has been shown to play a role in both the ER UPR and the mitochondrial UPR, which collectively integrates into a comprehensive stress response pathway referred to as the integrated stress response (ISR) [22]. The ER and mitochondria establish intricate structural and functional networks, known as mitochondria-associated ER membranes (MAM), which play a crucial role in maintaining cellular homeostasis and determining cell fate in diverse pathophysiological conditions [35]. Subcellular fractionation studies revealed that PERK is located within the MAM [36]. This unique subcellular localization strategically enables PERK to effectively coordinate the regulation of these two organelles in response to cellular stress. In line with this, PERK signaling has been documented to regulate various aspects of mitochondrial proteostasis and function, including SIMH during ER stress. During ER stress, PERK classically phosphorylates elF2α, leading to a global reduction of translation while concurrently promoting the expression of the transcription factor ATF4, which subsequently induces the expression of genes necessary for restoring homeostasis and ER function. The activation of PERK also enhances mitochondrial quality control mechanisms under stressful conditions by inducing the expression of crucial mitochondrial chaperones and proteases through intricate signaling pathways, either involving or extending beyond the classical PERK-elF2α-ATF4 pathway [22]. Through these pathways, PERK activation stimulates both mitochondrial biogenesis and autophagy processes, leading to the renewal and maintenance of a healthy mitochondrial network [22]. In addition, stress-activated PERK also enhances the formation of mitochondrial cristae and promotes the assembly of respiratory supercomplexes, thereby significantly augmenting cellular ATP-generating capacity [22]. Lastly, though the precise mechanism remains elusive, it is evident that PERK mediates mitochondrial hyperfusion following ER stress. This PERK dependent SIMH requires PERK-regulated elF2α phosphorylation and relies on the involvement of both SLP-2 and YME1L. Further, PERK-dependent SIMH takes place independently of ATF4, thus PERK-dependent SIMH is not a result of canonical ATF4-mediated gene expression during the PERK arm of the UPR [37]. Following ER stress, it has been shown that SIMH-incompetent cells exhibit premature fragmentation, a decrease in respiratory capacity, and may show increased cellular sensitivity to ER stress compared to SIMH-competent cells [37]. Thus, PERK mediated morphological remodeling of mitochondria plays a crucial role in the cellular response to ER stress.

Accumulation of misfolded and unfolded proteins within cells is a common pathological hallmark of neurodegenerative diseases. These protein aggregates can induce ER stress conditions, subsequently activating the UPR signaling pathways, wherein the PERK arm of the UPR has been particularly implicated [38]. As mentioned above, the UPR may play a proadaptive role under mild to moderate ER stress. However, in cases of severe or prolonged/excessive ER stress, the UPR may fail to restore homeostasis. In these cases, excessive stress leads to irreversible mitochondrial fragmentation and the activation of apoptotic signals, ultimately resulting in cell death. As a result, ER stress is considered a potential contributor to neurodegeneration [39]. Consistent with this, activation of the UPR has been associated with the onset of Parkinson’s disease [40]. On the other hand, inhibition of PERK has demonstrated beneficial effects in mouse models of Parkinson’s disease [41,42]. Though the exact relationship between cellular ER stress responses and neurodegeneration is not entirely clear, targeting the UPR has emerged as a promising therapeutic approach in the treatment of neurodegenerative diseases [43–45].

## METABOLIC STRESS

### Amino-Acid Starvation

The first report on the induction of mitochondrial hyperfusion by metabolic stress were obtained by replacement of mammalian cell culture media with Hank’s Buffered Salt Solution (HBSS) or in the tissue of fasted mice [23,46,47]. Starvation induced mitochondrial hyperfusion is a reversible process that protects mitochondria from starvation-induced autophagic engulfment, sustains ATP levels, and preserves cell viability during periods of nutrient deprivation [23,47]. The starvation-induced SIMH, akin to the CHX-induced mitochondrial hyperfusion [18], was found to be dependent on Mfn1 and OPA1, while being independent of Mfn2 and BAK/BAX. However, in contrast to CHX, which induced SIMH without impairing the activity of Drp1 [17], the mitochondrial hyperfusion induced by starvation is attributed to the inhibition of mitochondrial fission through phosphorylation of Drp1 at Ser637 by Protein Kinase A as well as dephosphorylation of Drp1 at Ser616 [47]. These two serine residues appear to exert contrasting effects on the processes of mitochondrial fission and fusion upon phosphorylation.

Phosphorylation of Drp1 at Ser616 promotes the occurrence of mitochondrial fission, whereas phosphorylation at Ser637 inhibits both enzyme activity and translocation of Drp1 to mitochondria, thereby impeding mitochondrial fission (for review see [48].)

It has been observed that the regulation of starvation-induced hyperfusion response occurs at multiple levels. In brief, following the initiation of SIMH, replacing cell culture media with HBSS supplemented with glucose, FBS, or insulin does not inhibit mitochondrial hyperfusion, while the inclusion of glucose and FBS even accelerate its progression [46]. Furthermore, in these early studies, it was observed that glucose or FBS deprivation alone is not sufficient to induce hyperfusion [47]. Interestingly, complete culture media lacking in amino acids is adequate for inducing SIMH [46,47], and the mere removal of glutamine alone can trigger hyperfusion [47]. In support of this finding, starvation media supplemented with either glutamine alone or essential amino acids (EAA) with glutamine, regardless of the absence of FBS and/or essential amino acids, effectively prevents the occurrence of SIMH. This finding suggests that a nitrogen-source deficiency can effect mitochondrial fusion [46,47]. Although treatment with EAA and glutamine may prevent the onset of SIMH, administration of EAA and glutamine does not possess the ability to reverse existing mitochondrial hyperfusion [46]. This suggests that the induction and maintenance of hyperfusion under starvation conditions are regulated independently.

Surprisingly, a recent study demonstrated that the addition of a combination of glutamine, leucine, and arginine (QLR) to amino acid-free starvation media significantly enhances mitochondrial fusion compared to that induced by complete amino acid deprivation [49]. It was also found that addition of QLR to DMEM does not induce any mitochondrial hyperfusion, suggesting that QLR-dependent mitochondrial hyperfusion requires the depletion of other amino acids. The data presented in this study also suggest that, consistent with previous studies, nutrition-starvation induced mitochondrial fusion is dependent on Mfn1 and Opa1. However, the involvement of Drp1 inactivation varies depending on cell type. Nevertheless, regardless of the cell type, Drp1 inactivation does not contribute to QLR-promoted mitochondrial hyperfusion [49]. Despite the fact that QLR strongly inhibits autophagy by activating mTORC1 [50], QLR-induced mitochondrial hyperfusion occurs independently of mTORC1. One possible mechanism for the promotion of mitochondrial hyperfusion by QLR is its incorporation into the catabolic pathway, including the tricarboxylic acid (TCA) cycle, leading to purine and nucleotide biosynthesis. This incorporation potentially increases GTP levels and subsequently stimulates Mfn1-mediated fusion of the outer mitochondrial membrane, thereby promoting stronger hyperfusion [49]. In another recent study, it was revealed that starvation-induced hyperfusion in COS7 African green monkey fibroblast-like cells is promoted by Mitochondrial Carrier Homolog 2 (MTCH2) in a manner dependent on Lysophosphatidic acid (LPA), a product derived from de novo lipogenesis [51].

### Glucose Starvation

The studies investigating the effects of glucose deprivation on mitochondrial dynamics have produced inconsistent findings. While some studies reported that deprivation glucose alone does not induce significant mitochondrial hyperfusion [47,49], another group has shown that glucose starvation induces SIMH [52]. The contradictory findings necessitate additional investigation and clarification to ensure a definitive resolution. Nevertheless, the induction of SIMH through glucose deprivation leads to a reduction in reactive oxygen species (ROS) production, while mitochondrial hyperfusion induced by glucose withdrawal occurs via a distinct pathway compared to amino acid deprivation [52]. Glucose-withdrawal SIMH is believed to result from increased fusion following deacetylation of Mfn1 at Lys222 by histone deacetylase 6 (HDAC6). This assumption is further supported by findings of HDAC6-dependent mitochondrial hyperfusion in the muscle tissue of fasted mice [52].

The central nervous system requires a disproportionately high amount of energy to function, and adaptation of both neurons and glial cells to various metabolic conditions plays a crucial role in maintaining the overall health of the nervous system (for review see [53,54]). In addition, starvation induces autophagy, which itself has a strong association with neurodegenerative disease (as reviewed by [55–57]). As discussed above, starvation induced mitochondrial hyperfusion protects against starvation induced autophagy, in turn preserving cell viability and sustaining ATP production during nutrient deprivation [23,47]. As such, starvation induced SIMH is an exciting new area of research with interesting potential for the study of neurodegenerative disease.

## DNA DAMAGE AND REPAIR

The vulnerability to damage is an inherent characteristic shared by all biological macromolecules, including lipids, proteins, and nucleic acids. Among them, the damage to a cell’s genomic DNA is particularly pronounced due to its indispensable role as the blueprint for protein synthesis, as well as being irreplaceable through resynthesis. Thus, cells have developed a sophisticated response network known as the DNA damage response (DDR), which functions to ensure efficient coordination of DNA damage detection, signal transduction, and repair mechanisms. Neurons are particularly vulnerable to the accumulation of DNA damage due to their high energy demands, increased transcriptional activity, and long lifespan [58].

Consequently, a growing body of evidence has implicated both DNA damage and impaired DNA repair in various neurodegenerative disorders [59,60]. The stress resulting from DDR is referred to as “genotoxic stress”. This genotoxic stress not only induces genome instability but also transiently triggers mitochondrial hyperfusion [61]. In the name-sake SIMH paper it was found that UV-irradiation induces SIMH [17]. Although it has been demonstrated that UV-irradiation causes DNA damage and activates DNA repair pathways [62], the question of whether the mitochondrial hyperfusion occurs in response to DNA damage or as a consequence of these repair pathways remains unknown. More recently, it has been shown that DNA damage induced by Phleomycin, peroxide, or Methyl methanesulfonate causes mitochondrial hyperfusion [61]. The induction of DNA damage triggers the activation of the DDR pathway in a manner dependent on mitochondrial fusion, as it has been observed that DNA repair is hindered when mitochondrial fusion is inhibited following DNA damage. Furthermore, it has been discovered that the mitochondrial fusion proteins Mfn1/Mfn2 interact with Sab and JNK, and these interactions are essential for the activation of JNK and subsequent ATM-mediated DNA repair [61].

## OXIDATIVE STRESS

The early investigations on mitochondrial hyperfusion induced by oxidative stress were conducted using isolated mitochondria in vitro. Treatment of isolated mitochondria with oxidized glutathione (GSSG), a crucial indicator of cellular oxidation, or peroxide resulted in the fusion of mitochondria [63]. Furthermore, elevated levels of GSSG in HeLa cells also induce the hyperfusion of mitochondria, which is attributed to disulfide-mediated oligomerization of mitofusin [63]. Subsequent reports have demonstrated that oxidative stress induces hyperfusion in differentiated mouse C2C12 myotubules by inhibiting glutathione synthase [64]. This hyperfusion in C2C12 myotubules is associated with increased sarcoplasmic reticulum (SR)-mitochondrial contacts (MAM) and altered calcium handling. Furthermore, culturing cells in oxidative media, where glucose is replaced with either galactose or acetoacetate (in cell media supplemented with dialyzed FBS), induces mitochondrial hyperfusion [65]. Replacement of glucose with galactose or acetoacetate is subsequently accompanied by an increase in oxygen consumption, which occurs independently of mitochondrial hyperfusion. Further, it is likely that the oxidative-media induced hyperfusion results from oxidative phosphorylation (OXPHOS) stimulated processing of Opa1 at the S2 site by YME1L driving inner-membrane fusion, while outer membrane fusion remained insensitive to OXPHOS-stimulation. The results also demonstrate that the hyperfusion induced by oxidative-media is independent of OPA1 cleavage at S1 by OMA1, as well as deacetylase activity. This suggests a distinct mechanism from glucose-starvation SIMH (discussed in the metabolic stress section) [65,66]. Oxidative stress has been implicated in neurodegenerative disease and neuroinflammation [67], and these new findings establish a connection between oxidative stress with mitochondrial dynamics.

## SMOKING/VAPING

Oxidative stress occurs when there is an imbalance between the generation and removal of reactive oxygen species (ROS) in cells and tissues due to their excessive production or inadequate detoxification. While ROS are primarily produced during normal oxygen metabolism, various environmental stressors like UV radiation, pollutants, heavy metals, and cigarette smoking significantly contribute to their formation [68,69]. Several studies have provided evidence on the impact of both cigarette smoke (CS) and e-cigarette smoke on mitochondrial function and health (reviewed by [16]). In brief, exposure to cigarette smoke extracts (CSE), electronic cigarette liquids/aerosols, and the residual particles left after smoking (known as thirdhand smoke or THS) induces mitochondrial hyperfusion [70–72]. This phenomenon has been observed in mouse epithelial cells as well as mouse neuronal stem cells, which are particularly sensitive to these toxins. This SIMH was considered a transient survival response accompanied by enhanced metabolism, increased ATP production, increased mitochondrial membrane potential, decreased mitophagy, and elevated ROS generation [71,72]. The observed effect was specific to mild or moderate levels of toxins, as high or prolonged exposure result in reduced cell proliferation and cell death [70,71]. The molecular mechanisms underlying this hyperfusion phenomenon remain largely unknown; however, it has been associated with upregulated expression of Mfn2 and downregulated levels of Fis1 mRNA. It is worth noting that exposure to CS has resulted in observations of either mitochondrial fragmentation [73] or elongated swollen mitochondria [74] in bronchial epithelial cells. Additionally, a study demonstrated that CSEinduced mitochondrial fragmentation is associated with the upregulation Drp1, while downregulating Mfn2, in airway smooth muscle cells [75]. These apparently contradictory findings may be attributed to variations in cell types and treatment conditions [70].

## DISEASE AND PHYSIOLOGY

Several models of physiology or disease have demonstrated the crucial role played by mitochondrial hyperfusion in the progression of phenotypes or as a cellular response promoting survival. This phenomenon has been observed in various conditions, including neurodegenerative diseases, aging, hypoxia, arrhythmia, and chronic kidney disease. Further investigation into the hyperfusion response and identification of the specific SIMH pathway activated in these diseases may significantly contribute to our understanding and subsequent treatment of these conditions.

### Neurodegenerative Disease

In the last decade, there has been a significant advancement in our understanding of the role of mitochondrial dysfunction in human pathologies. Although best known for their central function in energy production, mitochondria also have important roles in regulating apoptosis, calcium handling, innate immunity, and phospholipid synthesis. These diverse functions make mitochondria important for all cell types, but accumulating evidence suggests that cells with high energy demands, such as neurons are particularly susceptible to mitochondrial dysfunction. The pathogenesis of neurodegenerative disorders is closely associated with a profound impairment of mitochondrial functions, which can be triggered by various stress conditions such as oxidative and metabolic stress, defects in protein folding or import, and alterations in mitochondrial DNA. In response to these challenges, cells employ coordinated mechanisms of mitochondrial quality control, including mitochondrial fusion and fission as well as mitophagy, to maintain the homeostasis of mitochondria. Dysregulation of these mechanisms, such as excessive mitochondrial fission, downregulation of mitochondrial fusion, and a reduction in mitophagy caused by mitochondrial hyperfusion, are observed in neurodegenerative disorders, such as in PD [76], AD [77,78], Huntington [79,80], and Prion disease [81]. A decrease in the levels of mitofusin and Opa1 protein have been observed in brain tissue samples obtained from patients with Alzheimer’s disease (AD) [77,78], Huntington’s disease (HD) [80], and amyotrophic lateral sclerosis (ALS) [82]. Furthermore, it has been demonstrated that mutant huntingtin activates Drp1 through direct interaction [79]. On the other hand, the findings of a recent study suggest that a patient presenting with psychomotor developmental delay, global hypotonia, and severe ataxia resulting from axonal sensory neuropathy is most likely attributed to mitochondrial hyperfusion triggered by Drp1 mutations [83]. Therefore, it is likely that an imbalance in mitochondrial fusion and fission contributes to pathogenesis of these pathological conditions. The concept that both excessive mitochondrial fission and dysregulated mitochondrial hyperfusion can contribute to neuropathy is further supported by studies demonstrating that mutations in the fusion factor Mfn2, which either impair fusion or maintain fusion capacity, could lead to Charcot–Marie–Tooth disease type 2A (CMT2A) [84]. Furthermore, utilizing Drosophila models, it has been clearly shown that both dominant negative and dominant active forms of mitofusin can induce CMT2A-associated abnormalities, thereby proposing for the first time that excessive mitochondrial fusion drives the pathogenesis of CMT2A in a substantial number of patients [85]. Additionally, employing both in vivo and in vitro systems, it has been reported that knockout of Drp1 in neuronal cells leads to elongation of mitochondrial tubules, accumulation of mitophagy markers, and neurodegeneration [86]. This is most likely attributed to impaired mitophagy and inefficient removal of damaged mitochondria since mitophagy has been proposed to play crucial roles in turnover of dysfunctional mitochondria [87]. In addition to contributing to the pathogenesis, mitochondrial hyperfusion may also serve as a transient protective mechanism against aging and neurodegeneration [17,37,88].

### Aging and Senescence

One of the initial models implicating mitochondrial hyperfusion as relevant to disease and physiology was presented by Yoon et al. 2006, approximately three years prior to the name-sake SIMH paper [17,89]. This group showed induction of cellular senescence using the iron chelator deferoxamine, peroxide, or growth factor TGF-b1 leads to mitochondria hyperfusion. Furthermore, aged human diploid fibroblasts (HDFs) with a population doubling time of 90-94 days also exhibit hyperfused mitochondria. In these models, hyperfusion is attributed to decreased expression of Fis1, and overexpressing exogenous Fis1 inhibits both hyperfusion and senescence [89]. Moreover, inhibition of mitochondrial fission by the expression of dominant negative Drp1-K38A or Fis1 lacking a transmembrane domain (Fis1DTM) is sufficient to induce cellular senescence. Though senescence is characteristically difficult to generalize and is highly cell-specific, compelling evidence suggests that cellular senescence, as a vital biological process, plays a pivotal role in driving both the ageing process and neurodegenerative diseases (reviewed by [90]). Given the correlation between mitochondrial regulation and neurodegenerative disease, this may provide an exciting new avenue for neurodegenerative research, as aging is one of the most important risk factors for neurodegenerative diseases [90].

### Hypoxia and Acidosis

In a study on hypoxia and the resulting extracellular acidification, it was shown that low pH levels (pH = 6.5) induce mitochondrial hyperfusion in mouse cortical neurons [91]. Intriguingly, this hyperfusion response is exclusive to post-mitotic cells, including cerebellar granular neurons, ex vivo hippocampal slices, and differentiated C212 myotubules. In contrast, several prolific cell lines do not display hyperfusion upon extracellular acidification. This acidosisinduced hyperfusion was found to be independent of hypoxic conditions, though acidosistrig- gered mitochondrial hyperfusion protects cells from hypoxia-induced cell death in a fusiondependent manner. Acidosis-SIMH further aids cell viability during hypoxic treatments by maintaining mitochondrial membrane potential and ATP production.

The hyperfusion response to acidosis is thought to be due to inhibition of mitochondrial fission.

In cultured cortical neurons, Drp1 demonstrates a preference for binding with Fis1 rather than MFF, and this interaction decreases during acidosis-like treatments [91]. Consequently, the process of fission is hindered, thereby facilitating uninterrupted mitochondria fusion. While the post-mitotic specificity of this hyperfusion response is of particular interest to studies of the nondividing neuron cells, further research is necessary to establish a correlation between hypoxia, acidosis, and neurodegenerative disorders.

### Arrhythmia

Arrhythmia refers to the abnormal conduction, frequency, rhythm, and origin of electrical impulses in the heart. Atrial Fibrillation (AF), the most common form of sustained cardiac arrhythmia, is associated with high frequency electrical activity, impaired calcium handling, oxidative stress, and mitochondria dysfunction [64]. During sustained fibrillation, atrial myoblasts undergo adaptations to maintain calcium homeostasis known as atrial tachycardia remodeling (ATR). ATR alters calcium handling by the sarcoplasmic reticulum (SR), leading to SR dysfunction and ultimately sustained AF [64,92]. One way that calcium homeostasis is maintained is through mitochondria- associated membrane (MAM) contact sites between the SR and mitochondria. This tethering between SR and mitochondria is mediated by the protein Mfn2. Interestingly Mfn-2 also plays a role in various related functions, including mitochondria fusion, calcium homeostasis through the MAM, mitochondrial respiratory homeostasis, and the SR stress response [64]. The findings of one study indicate that fibrillatory stress leads to an increase in mitochondrial fusion and the recruitment of Mfn-2 to the mitochondria-associated membrane (MAM), suggesting a role of Mfn-2 and MAM in arrhythmogenesis [93].

The link between ATR mediated AF, MAM-mediated SR calcium handling, oxidative stress, and mitochondria dysfunction indicate that mitochondrial stress responses may play a role in atrial fibrillation and atrial arrhythmogenesis. Indeed, it has been shown using differentiated C2C12 mouse myoblasts that treatment with high-frequency electric field stimulation (EFS) (used as a model of AF-stress) leads to an increase in mitochondrial fusion, increase in MAM contact sites, altered calcium handling, and a hyperfused mitochondrial network. This adaptive response, however, is overridden by mitochondrial fragmentation in cases of persistent stress, leading to the initiation of mitophagy or cellular apoptosis. Further, differentiated C2C12 myoblasts undergoing oxidative stress (induced by the inhibition of reduced glutathione (GSH) synthesis) also showed an increase in MAM contact sites, altered calcium handling, and a hyperfused mitochondrial network [64]. These results demonstrate that there is an intimate link between the SIMH stress response and AF-associated stress, however more work needs to be done to determine how SIMH affects atrial arrhythmogenesis and the role of mitochondria in AF.

### Chronic Kidney Disease

In a model for chronic kidney disease (CKD), it was shown that the toxin p-cresyl sulphate (PCS), which accumulates in CKD patients and is particularly difficult to remove through hemodialysis, induces mitochondrial hyperfusion in cardiomyoblasts [94]. This hyperfusion is accompanied by an increase in oxygen consumption and mitochondrial respiration, enhanced mitochondrial biogenesis, and a slight decrease in mitochondrial membrane potential. It was observed that PCS-induced SIMH depends on AMPK (AMP-activated protein kinase) activation through phosphorylation at Thr172 upon PCS treatment. This model is of particular importance to CKD as cardiac remodeling is associated with CKD. This remodeling places patients at higher risk of cardiovascular disease, which is the major contributor to the exponentially elevated mortality risk as kidney function deteriorates [94].

## PERSPECTIVES AND CONCLUSIONS

**Figure 3.**
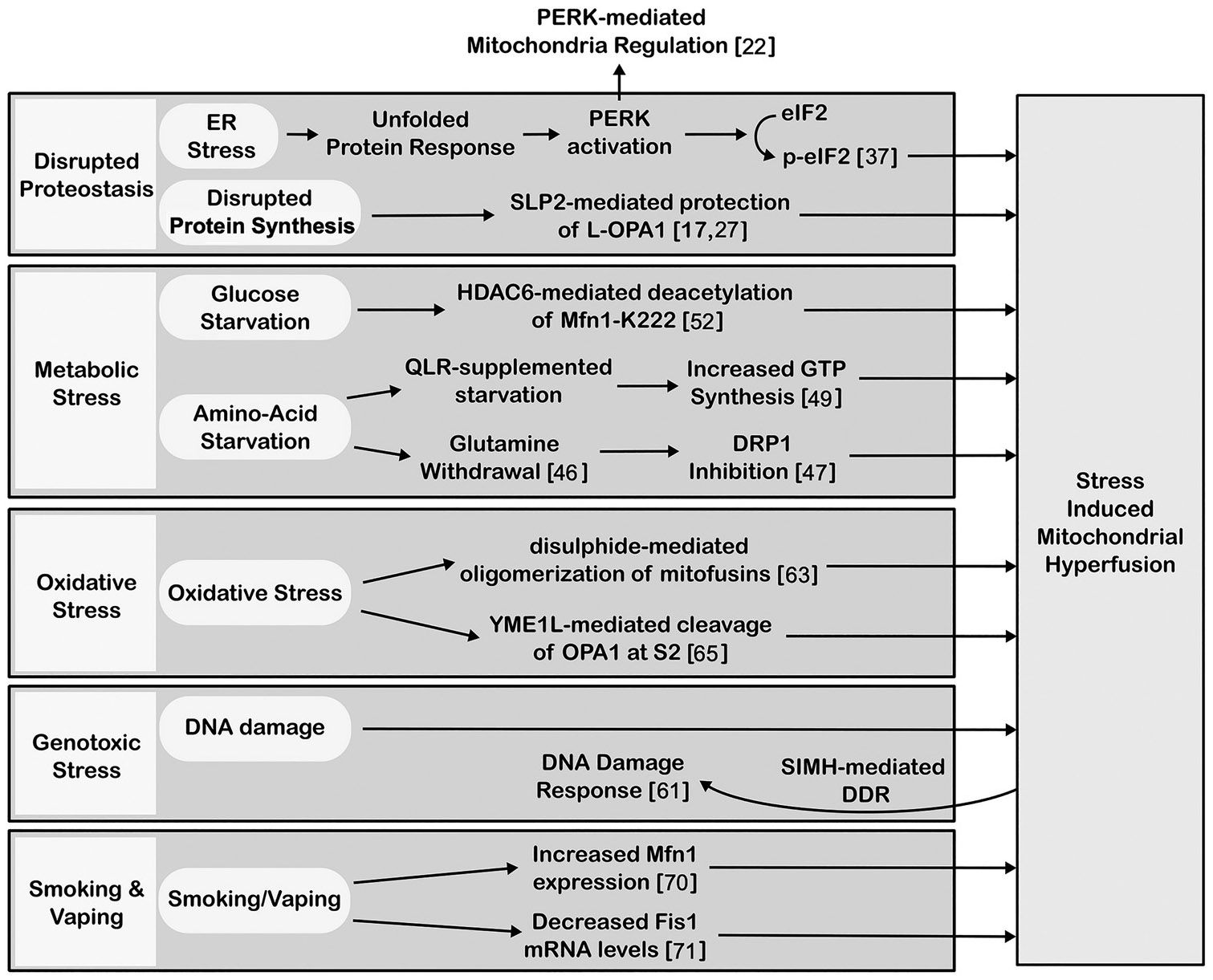
The diagram illustrates an overview of the stressors that trigger SIMH and their respective mechanisms It is evident that different types of stress activate diverse pathways, ultimately leading to the development of SIMH

The processes of mitochondrial fusion and fission, collectively referred to as “mitochondrial dynamics,” are highly coordinated phenomena. These dynamic alterations in shape and size play a pivotal role in the distribution of mitochondria, mechanisms for quality control, allowing mitochondria to adapt their morphology in response to energy demands and cellular conditions under normal circumstances as well as during stress or disease states. Inadequate response to these stressors may result in the accumulation of dysfunctional mitochondria due to failure to compensate for damage through fusion and eliminate damaged mitochondria through fission. Since the term stress-induced mitochondrial hyperfusion (SIHM) was coined by Tondera et al., [17], an increasing interest in SIMH has emerged due to research indicating that mitochondria can respond to stressful conditions by extensively fusing together, thereby forming a highly interconnected network. This process is believed to serve as a protective mechanism for sustaining cellular function and potentially preventing cell death. Thus, the stress-induced fusion of mitochondria holds significant therapeutic potential due to its capacity for maintaining mitochondrial health through the redistribution of healthy mitochondrial components, repair of damaged mtDNA, and effectively mitigating damage caused by stress. This mechanism potentially offers protection against various diseases associated with mitochondrial dysfunction, including neurodegenerative disorders, metabolic diseases, and heart failure by promoting cellular resilience under stressful conditions. [5,95,96]. However, as depicted in Figure 3, it is evident that different types of stress induce SIMH through distinct pathways. Therefore, one of the primary challenges in the field of SIHM lies in comprehending the precise mechanisms by which stress triggers fusion, particularly how diverse forms of stress can differentially impact the fusion process and effectively target these pathways to mitigate stress-induced damage while maintaining optimal mitochondrial dynamics within intricate cellular environments. While fusion is generally acknowledged as a protective response to stress, by facilitating the merging of damaged mitochondria with healthy ones, excessive fusion can disrupt cellular function and give rise to detrimental effects and disease progression [96–98]. Therefore, these potential drawbacks associated with SIHM pose another challenge in this field, underscoring the importance of identifying the signaling pathways that regulate beneficial versus excessive fusion under stressful conditions.

## List of abbreviations:

SIMH: Stress induced mitochondrial hyperfusion
ND: Neurodegenerative Disease
ER: Endoplasmic reticulum
SR: Sarcoplasmic reticulum
MAM: Mitochondria associated membrane
UPR: Unfolded protein response
ROS: Reactive oxygen species
CHX: Cycloheximide
Mfn: Mitofusin
OPA1: Optic atrophy 1
MFF: Mitochondrial fission factor
Fis1: Mitochondrial fission protein 1
Drp1: Dynamin related/-like protein 1
AMPK: AMP-activated protein kinase
